# Where an Explanation Seems Necessary

**Published:** 1902-02

**Authors:** 


### WHERE AN EXPLANATION SEEMS NECESSARY.

The fifty-two dental colleges in this country represent the elements in the dental profession that are constantly active in promoting its interests, intellectually and professionally, and the Association of Dental Faculties, which is composed of these fifty-two colleges as members, necessarily feels a deep interest in all that concerns its own welfare and that of the main professional body which it, in part, represents.

Several years since a committee was appointed by a resolution introduced into the Association of Faculties, to be called the Foreign Relations Committee. This was to be a standing committee, but the members were to be appointed or reappointed each year, and not elected as had been usual with the regular standing committees. The original intention was that this body should examine into the character of all foreign schools, and appoint advisory boards in all foreign countries to aid in giving information and, by endorsement of individual applicants, prevent unsuitable persons entering upon advanced standing in our American dental colleges. This praiseworthy position has been continued to the satisfaction of all concerned, and had it rested there, there could have been no just cause of complaint. This, however, was regarded by some as narrowing the work, and, limiting it to this, prevented future enlargement of its sphere of labor. The annual reports of this committee not only began to be voluminous, but constantly advised extension of its labor, and frequently assumed an authority not originally delegated to it. The Faculties complacently agreed to this, or quietly offered no direct opposition, until to-day the main body has been practically relegated to a subordinate position. The committee has so enlarged the scope of its labor since its first appointment that the majority of the main body have had difficulty in keeping in touch with its erratic movements. The result is that its reports have been repeatedly adopted without careful consideration. Each new presiding officer of the Faculties seems to deem it his duty to reappoint this committee, paying very little attention to the fact that the most important qualification for membership on it should be long attendance and active participation in the work of the body.

At the recent meeting of this Association at Milwaukee, the work of this Foreign Relations Committee occupied much of the important time of the Faculties. It grew eloquent in detailing the facts accumulated during the year, and the chairman forcibly advocated the importance of the Association entering actively into the arena of legal prosecutions of fraudulent colleges. The work of Consul Worman was enthusiastically portrayed, until the members became equally enthusiastic and voted an assessment of fifty dollars upon each of the colleges, and some few increased this to one hundred dollars. Consul Worman was naturally well satisfied with this, as he felt that at last some besides himself would help to bear the heavy expense to which he had voluntarily subjected himself, in collecting testimony to be used in prosecuting dealers in fraudulent diplomas in the State of Illinois. He remained over in Chicago after the meeting, incurring heavy obligations under the supposition that between the Faculties and the National Dental Association some provision would be made to reimburse him for the outlay. It is to be hoped that this has ere this been done, but up to a recent period nothing had been attempted in this direction.

The Foreign Relations Committee met at Chicago, at the Palmer House, on November 9, 1901, to consider the complex questions connected with and growing out of the expansion of the committee's work. This necessarily involved travel for its members, with one or two exceptions, of from five hundred to a thousand miles at a heavy expense, which must eventually be met by the colleges. The result of this conference is embodied in a circular issued at Buffalo, November 15, 1901. This circular is addressed "To the Deans and Representatives of the Dental Colleges Members of the National Association of Dental Faculties." The writer of this has been a delegate to represent an important college since he assisted in organizing the Faculties in 1884, but for some unexplained reason he was not honored with a circular. Possibly it may have been lost in the mails. He, however, secured a copy and extends his thanks thus publicly to the kind donor for the service rendered.

It is not an uncommon occurrence for committees and associations to be inconsistent in their rulings, but it has not often been the writer's experience to find a committee travelling a thousand miles with a single object in view, perpetrating a series of resolutions and other acts thoroughly inconsistent with each other and in direct opposition to the best interests of the association the said committee was supposed to represent.

From a series of eight resolutions passed at that meeting, the two following are quoted, italics ours:

"*Resolved,* That in the opinion of the Foreign Relations Committee, *while it should extend its moral support to every reform movement, it is not the function of the National Association of Dental Faculties to prosecute violations of the State dental laws or irregular acts of State dental examining boards.*"

The fifth resolution reads as follows:

"*Resolved, That the Foreign Relations Committee, under the power granted by the National Association of Dental Faculties,*

*will authorize the prosecution of fraudulent colleges whenever and wherever their existence is demonstrated to them."*

It is presumed the committee draws a wide distinction between violators of State dental laws and fraudulent colleges, but to the average mind the college that issues fraudulent diplomas is a violator of dental law. It would hardly seem necessary to undertake such an expenditure of time, money, and travel to frame such absurdly antagonistic resolutions.

The committee, after thus laboring and arranging to pay certain expenses in a certain way, called in consultation two eminent citizens of Chicago and one in the same city representing the State Examining Board, supposed at this period to have been reformed and purified. It is stated that when the gentlemen met with the committee, "each approved the action taken and accepted positions as members of the Prosecuting Committee, Dr. J. N. Crouse being designated as chairman. . . . The active co-operation of Consul Worman and the members of the Foreign Advisory Boards in the collecting of necessary testimony was assured.'"

The Foreign Relations Committee has, therefore, placed the whole matter of service in the hands of three parties, two of whom have not the slightest connection with the Association of Faculties, and the third is on record as openly opposing this body before the Illinois State Dental Society, and has never, to the writer's knowledge, been an active participant in its proceedings. The most prominent on this prosecuting committee, and who will be expected to take the active part in the prosecutions, has never omitted an opportunity to denounce dental colleges and the Association of Faculties, and in July, 1901, he took occasion, just prior to the meeting of the Association of Dental Faculties at Milwaukee, to denounce this body in unmeasured terms. That such a person should be appointed to represent the Faculties passes the writer's comprehension. The proceedings of this Foreign Relations Committee, as detailed in this circular, are humiliating to the last degree.

The allusion to Consul Worman and to the Foreign Advisory Boards may be based on correct information, but from what the writer knows of the way Consul Worman's report and voluminous testimony has been treated, it is extremely unlikely that he will labor very assiduously on behalf of the committee or the legal authorities of Illinois.

The next meeting of the Association of Dental Faculties is some months away, but it does seem that in the mean time the members should carefully consider the advisability of either reorganizing this Foreign Relations Committee or removing it entirely from the list of standing committees of this body. It is, at present, a menace to the best interests of that organization, and is gradually, but surely, submerging this educational association into a sea of legal quarrels and endless expense, the end of which no man has sufficient prescient knowledge to determine, or against which to provide means of protection.

